# Neuroendocrinology of a Male-Specific Pattern for Depression Linked to Alcohol Use Disorder and Suicidal Behavior

**DOI:** 10.3389/fpsyt.2016.00206

**Published:** 2017-01-03

**Authors:** Andreas Walther, Timothy Rice, Yael Kufert, Ulrike Ehlert

**Affiliations:** ^1^Clinical Psychology and Psychotherapy, University of Zurich, Zurich, Switzerland; ^2^Department of Psychiatry – Child and Adolescent Inpatient Service, Icahn School of Medicine at Mount Sinai, New York, NY, USA

**Keywords:** male depression, alcohol use disorder, suicidal behavior, steroid secretion, polymorphism, methylation, stress reactivity, vulnerability

## Abstract

Epidemiological studies show low rates of diagnosed depression in men compared to women. At the same time, high rates of alcohol use disorders (AUDs) and completed suicide are found among men. These data suggest that a male-specific pattern for depression may exist that is linked to AUDs and suicidal behavior. To date, no underlying neuroendocrine model for this specific pattern of male depression has been suggested. In this paper, we integrate findings related to this specific pattern of depression with underlying steroid secretion patterns, polymorphisms, and methylation profiles of key genes in order to detail an original neuroendocrine model of male-specific depression. Low circulating levels of sex steroids seem to increase the vulnerability for male depression, while concomitant high levels of glucocorticoids further intensify this vulnerability. Interactions of hypothalamus–pituitary–gonadal (HPG) and hypothalamus–pituitary–adrenocortical (HPA) axis-related hormones seem to be highly relevant for a male-specific pattern of depression linked to AUDs and suicidal behavior. Moreover, genetic variants and the epigenetic profiles of the androgen receptor gene, well-known depression related genes, and HPA axis-related genes were shown to further interact with men’s steroid secretion and thus may further contribute to the proposed male-specific pattern for depression. This mini-review points out the multilevel interactions between the HPG and HPA axis for a male-specific pattern of depression linked to AUDs and suicidal behavior. An integration of multilevel interactions within the three-hit concept of vulnerability and resilience concludes the review.

## Introduction

Epidemiological studies suggest that women experience depressive disorders at two to three times the rate of men (1, 2). However, for men in Western countries, concomitant low levels of diagnosed depression alongside high rates of alcohol use disorders (AUDs) and suicide are reported (3, 4). Suicide and AUDs are more strongly intertwined within males than females (5). This finding is supported by a study showing that men are more likely to have elevated blood alcohol levels at the time of completed suicide (6). Furthermore, over 2% of traffic accidents are classified as road traffic suicides, of which are committed to around 90% by men, and AUDs are regarded as important risk factor (7, 8). Some consider AUDs and suicidal behavior to be dysfunctional coping mechanism products of depression (9), while others with supporting longitudinal data show instead a more complex bidirectional relationship between depression and AUDs (10). Together, these data raise the question of whether there exists a unique and potentially underdiagnosed male-specific pattern of depression that is linked with AUDs and suicidal behavior (3, 11).

KEY CONCEPT 1 Male-specific pattern of depression The male population generally has lower rates of depression compared to the female population; however, depression in men is associated with higher rates of alcohol dependence and suicide than the rates seen in women. These behavior patterns are thought to represent dysfunctional coping mechanisms in depression, thus creating a unique subcategory of patients.

The assumption of a male depression has generated much investigation in the field of psychiatry. These investigations are high yield in determining how best to reduce male AUDs and suicide rates, particularly in special populations of sociopolitical importance such as war veterans and others (12–14). Despite many efforts, to date, no simple neuropsychiatric model has been proposed to account for men’s increased AUDs and suicide rate relative to their lower rates of depression.

Emerging interest in the interplay between neuropsychiatry and endocrinology may yield an improved cross-disciplinary model to account for a male-specific pattern for depression, its interrelation with AUDs, and suicide. In this mini-review, we intend to examine evidence supporting an underlying neuroendocrine model for a male-specific pattern for depression linked to AUDs and suicidal behavior. Understanding the pathophysiology of a male-specific susceptibility to depression, AUDs, and suicidal behavior might enable the allied mental health fields to develop tailored and highly effective combined treatments consisting of psychotherapeutic and pharmacological interventions.

KEY CONCEPT 2 Cross-disciplinary model A model integrating scientific methods and evidence from both neuropsychiatry and endocrinology, to promote a better understanding of gender-specific clinical manifestations such as male-specific depression.

## Changes in Steroid Hormone Concentrations

Testosterone, the end product of the hypothalamus–pituitary–gonadal (HPG) axis, has been investigated extensively as putative biomarker of depression. Studies indicate that hypogonadal men are more likely to develop depression (15). Testosterone treatment has been shown to exhibit beneficial effects on mood in men (16, 17). These effects may be age-specific as low levels of testosterone seem to be associated with suicidal behavior in older men, while high testosterone levels might be associated with suicidal behavior in youth (18–20).

KEY CONCEPT 3 Hypothalamus–pituitary–gonadal axis Gonadotropin-releasing hormone is secreted from the hypothalamus and stimulates production of luteinizing hormone (LH) and follicle-stimulating hormone (FSH) in the pituitary gland. LH and FSH then stimulate production of estrogen and testosterone in the gonads. Testosterone tends to be lowered in specific subcategories of men with depression.

KEY CONCEPT 4 Hypothalamus–pituitary–adrenocortical axis Corticotropin-releasing hormone is secreted from the hypothalamus and stimulates the secretion of adrenocorticotropic hormone (ACTH) in the pituitary gland. ACTH then stimulates the production of cortisol in the adrenal glands. Cortisol tends to be elevated in patients with depression, which is considered a stress-related disease.

Despite these findings and a large body of literature demonstrating a beneficial influence of testosterone on well-being and depression in men (21–24), a recent review concludes that the study of men as a large heterogeneous population yields no consistent relationship between testosterone and mood (25). These studies may lead some to conclude that there is no association between testosterone, depression, and suicide attempts (26). However, statistically significant findings may emerge when examining specific subgroups of men where an association between low testosterone levels and depression is more pronounced. For example, treatment-resistant depressive men, men with major depression and comorbid human immunodeficiency virus infection, hypogonadal men, dysthymic men, and elderly men (>60 years) often have lower testosterone levels (25). These may be specific populations of special importance in exploring the relationship between testosterone to men’s mental health.

Additionally, depressed individuals appear to have a dysregulated and hyperactive hypothalamus–pituitary–adrenocortical (HPA) axis. HPA axis disturbances have been linked to the development and maintenance of depression (27). Depression is considered a stress-related disease with altered glucocorticoid receptor (GR) signaling and reduced glucocorticoid responsiveness (28, 29). Cortisol, the end product of the HPA axis, generally tends to be significantly elevated in depressed subjects (30). Higher circadian cortisol secretion patterns were also found in subgroups of depressed patients compared to healthy controls (31). However, several moderators need to be taken into account, when interpreting the literature on cortisol and depression including methodological (time of day, challenge tests, or body substrate), sample-related (age or symptom severity), and depression-subtype (melancholic, psychotic, or atypical depression) moderators (27). Highest cortisol concentrations in depressed compared to healthy individuals were found when assessing cortisol in the afternoon, using challenge tests, the use of blood or cerebrospinal fluid samples as compared to salivary or urine samples, older age and greater symptom severity/hospitalization status were given, and psychotic or melancholic depression was exhibited.

Other neuroactive steroids such as dehydroepiandrosterone (DHEA) and estradiol are also implicated in the onset and maintenance of depression. These agents may all have potential antidepressant effects. DHEA works concomitantly as a precursor hormone of testosterone or estradiol while also exerting independent effects on different body systems such as the HPA axis (32). Low DHEA levels have consistently been related to depressive symptoms (33). Moreover, the use of DHEA as an antidepressant therapy has shown some success (34). Similarly, low levels of estradiol have been associated with more depressive symptoms in men (35). Carrier and colleagues report concomitant testosterone and estradiol administration to exhibit antidepressant-like effects in male gonadectomized rats, suggesting that testosterone’s protective effect may be mediated, in part, by its aromatization to estradiol (36).

Similarly, in both, chronic alcohol-dependent patients and moderate chronic alcohol consumers reduced testosterone and DHEA levels and increased basal levels of cortisol are reported (37, 38). As alcohol intake contributes to HPA axis activation, chronic heavy alcohol use leads to chronic HPA axis activation accompanied by the loss of normal diurnal cortisol secretion pattern; this persists during withdrawal and is mostly reestablished after 1–4 weeks after abstinence (39). In contrast, alcohol intake inhibits the HPG axis and suppresses testosterone production (40). Testosterone suppression might further contribute to depressed mood leading to disproportionately high suicide rates seen in men with comorbid depression and AUDs (41). Reduced basal androgens and elevated glucocorticoids seem therefore to be a shared endocrine phenotype in male depression and AUDs.

## Genetic Risk Constellation

Genetic factors determine steroid secretion and action, while steroids regulate gene expression *via* intracellular receptor binding (42). For example, testosterone action is modulated by the CAG repeat length polymorphism in the X-chromosome-bound androgen receptor (AR) gene. It is proposed that longer CAG repeat length causes lower transcriptional activity of genes activated by testosterone binding (43). A longer (>23) and a shorter (≤20) than average amount of CAG triplets have been suggested as risk alleles (44). Longer CAG repeat length is associated with more depressive symptoms (45). Simultaneous assessment of the CAG repeat length, testosterone levels, and depressive symptoms showed that low testosterone levels were associated with depression in men with the shorter allele only (46). However, this finding was not replicated (43), and for boys, testosterone was negatively associated with depressive symptoms only when expressing the longer allele (44). In addition, reduced CAG repeat length was associated with increased craving symptoms but was not significantly different between AUD patients and healthy controls (47). The CAG polymorphism may thus be a mediator that warrants consideration in future studies.

KEY CONCEPT 5 Steroid secretion Different patterns of steroid secretion are associated with different clinical manifestations. For example, low circulating levels of sex steroids increase vulnerability for male depression, and this vulnerability is further increased by high levels of glucocorticoids.

KEY CONCEPT 6 Polymorphism Differences in the DNA sequence that account for the variation between different individuals. For example, longer CAG repeat has been associated with more depressive symptoms.

The Val66Met polymorphism in the brain-derived neurotrophic factor (BDNF) gene causing deficient BDNF protein secretion affects the neuroplasticity processes crucial for depression (48). Research reports an association between the Val66Met polymorphism and depression (49–52). Sex steroids were shown to increase BDNF protein levels in human neurons suggesting sex steroids to have additional protective effects against depression due to the promotion of neuroplasticity (53). Similar effects were reported for other recently developed antidepressant medications further underlining the importance of increasing neuroplasticity in depressed individuals (54). However, a meta-analysis including 28 studies questions the assumed beneficial effects of BDNF by postulating no association between genetic variants in BDNF and major depression (55). It is possible that BDNF affects depression only *via* the interaction with other polymorphisms or steroid secretion (56). In addition, in female suicide attempters HPA axis hyperactivity was associated with decreased BDNF (57). A trend toward decreased BDNF serum levels was also reported in AUD patients (58), though these results could not be replicated in another study (59). In sum, throughout the literature one encounters an inconsistent picture for the relationship between BDNF and depressive disorders, AUDs, and suicidal behavior.

More consistent findings have been reported for two common variants in the 5-hydroxytryptamine transporter-linked polymorphic region (5HTTLPR). Homozygous and heterozygous carriers of the short allele variant were found to be at increased risk of major depressive disorder. Notably, homozygous carriers of the short allele are also at increased risk for alcohol dependence (60), lending an interesting biological footprint to the association between AUDs, depression, and suicide in men. Examining the concomitant effects of another polymorphism (C1019G) from the serotonin receptor gene, 5HT1A and the Val66Met polymorphism of the BDNF gene revealed increased risk for depression when expressing both risk variants (61). In addition, carriers of the long allele with concomitant higher levels of testosterone showed lower cortisol secretion after threat indicating neuropsychiatric resilience for this combination in humans (62). A recent review on genetic association studies of suicidal behavior identified 5HTTLPR and BDNF among few others as most promising candidates (63). Male-specific endocrine factors may play a key role in this association.

Recently, a genetic variant within the gene encoding for FK506 binding protein 5 (FKBP5) was shown to be associated with major depression (64). FKBP5 is considered to regulate intracellular GR signaling (65). Furthermore, FKBP5 ablation in mice was shown to increase antidepressant behavior (66). As an example of the delicate system interplays, FKBP5 is a glucocorticoid-induced negative regulator of the GR, yet at the same time, it is a positive regulator of the AR. This concomitant action of FKBP5 suggests that it functions as a reciprocal modulator of glucocorticoid- and androgen-mediated physiology (65).

The gene NR3C1, which encodes the GR, a crucial element for modulation of HPA axis function, has been extensively examined with regard to depression. An *in vitro* mouse model showed testosterone treatment to downregulate NR3C1 expression linking androgens with NR3C1 inhibition (67). It has been shown that three polymorphisms (rs6198, rs6191, and rs33388) within the NR3C1 resulting in GR resistance are associated with major depression and the predominance of depression in the course of bipolar disorder (68). Three other single nucleotide polymorphisms [SNPs (*Bcl*I, N363S, and ER22/23EK)] of NR3C1 were also associated with the increased recurrence of depressive disorders (69). However, there is conflicting literature reporting a lack of association: a 4-year prospective study investigated 683 subjects with major depression in remission with regard to time until recurrence of a major depressive episode. GR polymorphisms (9β, ER22/23EK, *Bcl*I, TthIIIl, NR3C1-1, and N363S) were not associated with recurrence of depression (70). Another study investigating the association between polymorphisms in NR3C1 and suicide attempts in 597 affective disorder patients reports no difference between groups with and without a history of suicide attempts (71). Recent studies show SNPs in FKBP5 and NR3C1 to be associated with alcohol drinking and crucial for alcohol abuse interventions (72, 73).

In conclusion, these results indicate subgroup-specific effects of polymorphisms and interaction with other SNPs, and steroid hormone levels to be relevant for a specific pattern for depression linked to AUDs and suicidal behavior in men. Studies show an interchange between candidate genes for depression and those within the endocrine system relevant for circulating levels of androgens and glucocorticoids, which warrant further investigation.

## Epigenetic Risk Constellation

Examining potential gene–environment interactions, methylation studies for the aforementioned risk alleles were investigated with regard to a male-specific pattern for depression. To date, methylation of the AR promoter region was primarily investigated in relation to prostate cancer, where DNA hypermethylation of the AR promoter region occurs leading to AR downregulation (74, 75). As low levels of testosterone are associated with depression in men and hypermethylation of the AR promoter region reduces testosterone signaling and action at target cells, hypermethylation of the AR might be associated to neuropsychiatric manifestations in men, including depression, AUDs, and suicidal behavior.

KEY CONCEPT 7 Methylation The addition of methyl groups to DNA, usually resulting in inhibition of gene transcription.

Brain-derived neurotrophic factor has recently become the focus of methylation studies associated with depression. Several studies indicate promoter methylation of BDNF to be associated with major depression (76). BDNF promoter methylation was shown to be associated with cortical thickness in patients with recurrent major depression (77). Another study revealed that higher BDNF promoter methylation was associated with more depressive symptoms and increased risk of drug addiction (78). Therefore, BDNF promoter methylation might independently contribute to the etiology and maintenance of depression, AUDs, and suicidal behavior in men, and it may mediate the effects of methylation on endocrine system receptors and signaling.

Similarly, homozygous and heterozygous carriers of the short variant of the 5HTTLPR exhibit higher mean 5HTT methylation and have therefore a lower 5HTT expression in peripheral blood mononuclear cells (79). Higher 5HTT methylation, but not HTTLPR polymorphism, was associated with more stress reactivity in infant macaques (80). Patients with depressive disorder were also shown to have a higher mean methylation level of the 5HTT gene than healthy controls (81). However, in patients with alcohol dependence, no different 5HTT methylation pattern was found (82).

FK506 binding protein 5 and NR3C1 were shown to be associated with depression and suicide attempts (83, 84). Roy and colleagues report for a sample consisting predominantly of men (90%) with a history of substance abuse a significant interaction between childhood trauma and variants of FKBP5 to raise the risk of attempting suicide (84). Therefore, their methylation status was further examined with regard to depression, indicating independent effects on risk for depression establishment and severity (31, 83). A 10% higher methylation rate of FKBP5 intron 7 for individuals with a lifetime history of major depression compared to healthy controls was found (85). Maternal depression during pregnancy was associated with NR3C1 hypermethylation in the newborn (86). Early-life stress-induced methylation of the NR3C1 was associated with subsequent demethylation of FKBP5, which thereby links these two stress responsive genes *via* epigenetic alterations (87). Replication of these findings in exclusively male samples is needed to further elucidate the epigenetic profile of the investigated male-specific pattern for depression. However, combat veterans with diagnosed PTSD often suffer from comorbid depression and AUDs. An intervention study reported that in combat veterans, who were receiving psychotherapy, the methylation of the NR3C1 promoter pretreatment significantly predicted subsequent treatment outcome, while FKBP5 methylation increased with regard to treatment (88).

Taken together, the influence of the gene–environment interaction causes endocrine alterations in depression and AUDs, which are determined by both genetic variants and epigenetic profiles. Examining further genetic variants and epigenetic profiles would be highly interesting to further capture relevant alterations underlying the relationship between depression, AUDs, and suicide in men (89, 90).

## Changes in Endocrine Stress Reactivity

Patterns of stress reactivity, or the physiological response of the individual to psychosocial stressors, may also reveal insights into male-specific behavioral health that intertwine with endocrine functioning.

Numerous studies confirmed an increase in cortisol by exposure to acute psychosocial stress (28). A meta-analysis indicates HPA axis response to psychosocial stress of depressed subjects is similar to healthy controls. However, depressed subjects with high basal cortisol were found to have increased cortisol production and higher cortisol levels during psychosocial stress (91). Indeed, in numerous studies reduced responsiveness to glucocorticoids has been reported for depressed patients assessed *via* the combined dexamethasone-suppression/corticotropin-releasing hormone (DEX-CRH) test indicating impaired GR signaling (29). This is further supported by meta-analytic findings of higher cortisol levels during the recovery period in depressed patients compared to healthy controls, while cortisol secretion patterns during stress were similar (92). This is of particular interest for a male-specific pattern for depression, as men show generally higher HPA responses to psychosocial stress, and estradiol seems to exert buffering effects (93). In contrast, for individuals with AUDs, there are blunted HPA axis responses to psychosocial stress and exogenous CRH provocation (38, 94). Interestingly, in depressed patients with suicidal behavior, blunted responses to the DEX-CRH test were also found in comparison to depressed patients without suicidal behavior (95). The existing data point to a chronic hyperactive HPA axis with a blunted acute stress response for a male-specific pattern for depression linked to AUDs and suicidal behavior.

Recently, sex steroids were also shown to depict a stress-dependent rise (96). However, there are conflicting findings (97). In addition to cortisol, sex steroids might represent additional physiological markers for the stress reactivity after a psychosocial stressor (28, 96). Attenuated DHEA-S response during acute psychosocial stress has been demonstrated in healthy men perceiving stress at work and patients with burnout (98, 99). Studies on testosterone or estradiol with regard to depression and their stress reactivity to psychosocial stress are scarce. Decreased baseline plasma testosterone was seen in adult male rats after an immobilization stress (100), while another study reported an increase in plasma testosterone after immobilization stress (101). In the forced swimming test estradiol significantly increased, while no change was observed for testosterone in rats (102). Taken together, conflicting literature is reported with regard to depressive symptoms and the reactive secretion of sex steroids in response to psychosocial stress.

## Integration into the Three-Hit Concept of Vulnerability and Resilience

In conclusion, the three-hit concept of vulnerability and resilience, fully described elsewhere (88), offers a theoretical framework integrating the interactions of the HPA and HPG axis-related polymorphisms, methylation profiles, steroid secretion, and endocrine stress reactivity with regard to depression, AUDs, and suicidal behavior in men.

KEY CONCEPT 8 Three-hit concept of vulnerability and resilience A proposed model stating that stress in early life can affect behavioral adaptation to stress later in life. The first hit is genetic predisposition; the second hit is early-life environment, which programs phenotypes by epigenetic regulation; and the third hit is later-life challenges.

Genetic risk variants determine an initial vulnerability for depressive disorders, AUDs, and suicidal behavior in men. Hit one represents polymorphisms in the AR, BDNF, 5HTTLPR, FKBP5, and NR3C1, which independently increase the risk of developing depression, AUDs, and suicidal behavior in men. Following the common variant hypothesis, combinations of risk alleles such as a long variant (>23) of the CAG repeat length in the AR, a short variant of the 5HTTLPR, and one of the three polymorphisms of the NR3C1 (rs6198, rs6191, and rs33388) increase the risk for these conditions in men multiplicatively.

Early-life environment and its experience, representing hit two, such as adequate or inadequate nutrition, childhood traumas, and adverse childhood experiences, and optimal or lacking parent–child affective attunement and dyadic regulation create unique phenotypes *via* the interaction with multigenic input by epigenetic regulation. Epigenetic regulation of HPA or HPG axis-related gene sites help the developing organism adapt to altered environmental conditions. Hypermethylation or hypomethylation of key genes for the development of depression in men, such as the AR, BDNF, 5HTT, FKBP5, and NR3C1 constitute independent risk and resilience factors for depression, AUDs, and suicidal behavior and further interact with the prior described genetic predisposition. This interaction forms a differential susceptibility to later-life challenges.

**Figure 1:**
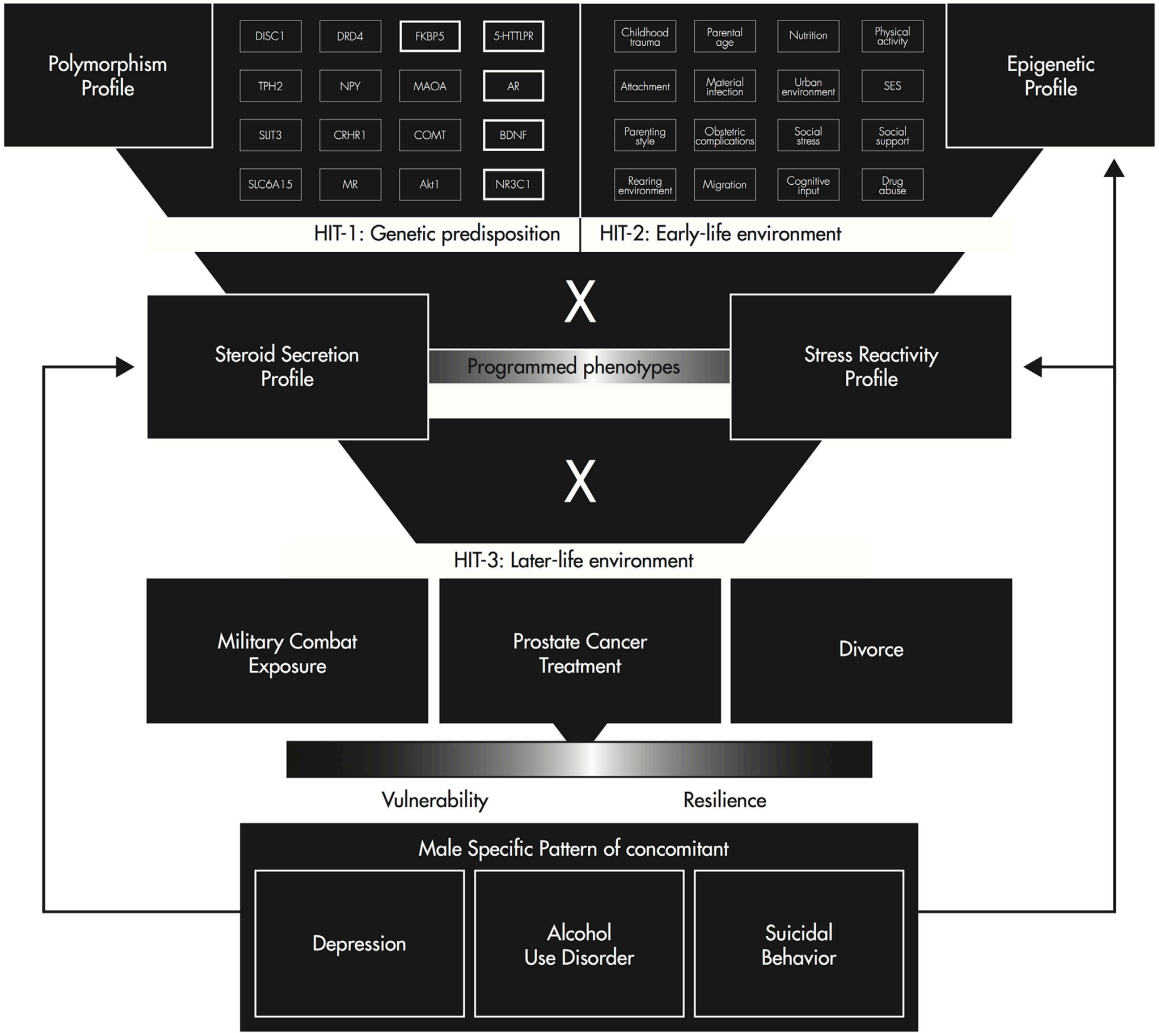
**Three-hit concept of vulnerability and resilience for a male-specific pattern of depression, alcohol use disorder, and suicidal behavior [adapted from Ref. (103)]**.

Later-life environment, such as exposure to trauma or major critical life events such as military combat, prostate cancer, or divorce, is considered hit three. As shown in Figure 1, depending on the interaction of programed phenotypes with later-life challenges, a man would either suffer from depression, AUDs, or suicidal behavior or he would develop mental resilience. Finally, the established pattern of depression, AUDs, and suicidal behavior influences steroid secretion, endocrine stress reactivity, and the epigenetic profile. Therefore, experimental human studies examining HPA and HPG axis activity and function in parallel also in response to stress are needed to untangle the complex interactions between the genetic predisposition, early-life environment, and later-life challenges underlying this male-specific pattern of depression, AUDs, and suicidal behavior.

## Limitations

Some limitations should be taken into consideration when interpreting the literature.

The reported studies examined biological differences between patients with depression, AUDs, suicidal behavior, and healthy controls and were not testing our proposed model specifically. Therefore, large-scale studies with male samples are needed to determine the actual overlap in depression, AUDs, and suicidal behavior using male-specific psychometric instruments as suggested by Rice and colleagues (104). We also cannot rule out that the results described here are partially confounded by a publication bias caused by non-publication of null results in this research area. A third limitation is that no restrictions were made with regard to inclusion of studies reporting hormonal data. Circadian rhythmicity, prior activities, intraindividual changes in subsequent days, huge interindividual differences in circulating concentrations, used body substrates, sampling techniques, and hormone-assays are potential confounding issues hindering the establishment of reference ranges. Finally, we examined only genetic, epigenetic, and endocrine data, while additional relevant areas such as immunological and functional neuroimaging data were beyond the purview of this mini-review.

## Conclusion

The high rate of AUDs and suicides juxtaposed to the low rate of diagnosed depression in men led to the assumption of an underdiagnosed male depression related to AUDs and suicidal behavior. We here provide evidence for overlapping neuroendocrine conditions underlying these disorders in men adding a biological perspective to theories on socialization and masculinity trying to explain the discrepancy of depression diagnosis in men and women (105). In men, depression, AUDs, and suicidal behavior seem to interact dynamically and to be associated with multiple biological risk factors such as decreased basal androgen and increased glucocorticoid levels, a blunted cortisol stress response and SNPs and hypermethylation/hypomethylation in candidate genes (AR, BDNF, 5HTTLPR, FKBP5, and NR3C1). Once established, these disorders further cause additional dysregulations in the HPA and HPG axes. Further research at the intersection of neuropsychiatry and endocrinology will advance our neurobiologically informed understanding of men’s mental health.

## Author Contributions

AW conducted a systematic review of the topics literature, which he integrated in a first draft of this mini-review. He subsequently reviewed further editing from coauthors. TR substantially contributed to the conception of the work, included further literature, and edited the first draft. YK critically reviewed the literature used, updated it, and edited a subsequent draft. UE critically reviewed the manuscript and edited it to its final form.

## Conflict of Interest Statement

The authors declare that the research was conducted in the absence of any commercial or financial relationships that could be construed as a potential conflict of interest. The reviewer NK and handling Editor declared their shared affiliation, and the handling Editor states that the process nevertheless met the standards of a fair and objective review.
